# Intracerebral Hemorrhage: Perihemorrhagic Edema and Secondary Hematoma Expansion: From Bench Work to Ongoing Controversies

**DOI:** 10.3389/fneur.2016.00210

**Published:** 2016-11-21

**Authors:** Manoj K. Mittal, Aaron LacKamp

**Affiliations:** ^1^Department of Neurology, University of Kansas Medical Center, Kansas City, KS, USA; ^2^Department of Anesthesiology, University of Kansas Medical Center, Kansas City, KS, USA

**Keywords:** intracerebral hemorrhage, secondary brain injury, perihemorrhagic edema, hematoma expansion

## Abstract

Intracerebral hemorrhage (ICH) is a medical emergency, which often leads to severe disability and death. ICH-related poor outcomes are due to primary injury causing structural damage and mass effect and secondary injury in the perihemorrhagic region over several days to weeks. Secondary injury after ICH can be due to hematoma expansion (HE) or a consequence of repair pathway along the continuum of neuroinflammation, neuronal death, and perihemorrhagic edema (PHE). This review article is focused on PHE and HE and will cover the animal studies, related human studies, and clinical trials relating to these mechanisms of secondary brain injury in ICH patients.

## Introduction

Intracerebral hemorrhage (ICH) is a medical emergency, which often leads to severe disability and death (1). ICH-related poor outcome is due to the combined effect of primary injury and secondary injury. In the primary injury, the hematoma cleaves or dissects neuronal tissue over several hours leading to the presenting symptoms. The loss of neurons within the hematoma is extensive; although some islands of preserved neurons may exist (2). Hematoma expansion (HE) stops due to tamponade effect of the surrounding tissue or clotting at the parent vessel. Due to the rapid onset, the primary injury of ICH is difficult to treat. The best way to minimize the injury from primary insult would be the preventative measures in high-risk individuals and early access to care once symptoms start. Primary injury is followed by secondary injury in the perihemorrhagic region over several days to weeks and provides a longer treatment window than the primary injury. In survivors of ICH, the total extent of remodeled brain tissue may exceed the volume of the initial hematoma. This excess volume of remodeling serves as an evidence of secondary injury. There are multiple factors that contribute to secondary injury after ICH, some of which may occur concurrently or in sequence. Secondary injury after ICH can be separated into two major types: rebleeding causing HE and the consequences of repair pathways along the continuum of neuroinflammation and neuronal death, including perihemorrhagic edema (PHE), elevated intracranial pressure (ICP), hydrocephalus, and brain atrophy. This review article is focused on PHE and HE, and will cover the animal studies, related human studies, and the clinical trials relating to these mechanisms of secondary brain injury in ICH patients. Although numerous animal studies have shown promising targets for therapy to reduce PHE and HE, we currently do not have any approved therapy for either. Practice guidelines published by the American Heart Association/American Stroke Association (AHA/ASA) in 2015 did not recommend any current therapy for PHE or HE (1).

## Perihemorrhagic Edema

Perihemorrhagic edema is often seen in ICH patients and causes mass effect on adjacent brain structures, elevation in ICP, hydrocephalus, or brain herniation often leading to clinical deterioration. The extent of PHE after ICH is variable and is governed by several mechanisms including the early effects of the Starling oncotic forces and the late effects of cell breakdown, neuroinflammation, and disruption of the blood–brain barrier (BBB). The early PHE occurs without disruption of the BBB (3, 4). PHE starts within 3 h of ICH onset and grows fastest in the first 3 days. PHE continues to grow for 7 days in rats (5) and for as long as 3 weeks in humans with peak between 2 and 3 weeks (4). Absolute PHE volume is linked to hematoma volume, low systolic blood pressure (BP), and high admission hematocrit (4). Admission partial thromboplastin time, baseline hematoma volume, history of hypertension, and earlier time from hematoma onset to CT are associated with higher relative PHE, defined as PHE per hematoma volume (4). PHE growth is associated with decline in neurological examination during hospitalization (6). Absolute, relative, and rate of increase in PHE are all related to death or dependency at 90 days (4, 7, 8). PHE-associated effect on clinical outcomes may be dependent on hematoma volume, as shown in three previous studies where PHE in setting of moderate size hematoma volume around 30 cc was associated with poor outcomes (9–11). Hematoma location in basal ganglion was also found to be associated with an increase in PHE-related adverse outcomes (10). The formation of post-ICH brain edema is related to direct clot retraction releasing serum, which causes hydrostatic pressure leading to early edema in the first 72 h (12). Delayed edema is secondary to neuroinflammation and oxidative stress (13) from a cascade of reactions triggered by red blood cell lysis, thrombin production, complement activation, and neuroinflammation, which in turn causes damage to the BBB (14–16). NXY-059, a free radical scavenger, was studied in comparison to placebo in 607 patients and showed no benefit on HE, PHE, or clinical outcomes (17). The animal studies done so far have helped to delineate these mechanisms and provided some data for therapeutic targets.

### Treatment of Perihemorrhagic Edema

#### Targeting Direct Clot-Related Hydrostatic Pressure

Direct effect of clot-related hydrostatic pressure can be minimized using clot removal and/or osmotherapy. We present here the bench to bedside studies supporting each of these two treatment options.

##### Surgery

Animal magnetic resonance imaging (MRI) studies have found that PHE corresponds to reduced perfusion in the perihemorrhagic area (18, 19), but does not represent cytotoxic edema. Hematoma evacuation in study animals led to normalization of this reduced perfusion (18, 20). The non-cytotoxic nature of PHE is further supported by the lack of effect of reductions in BP and cerebral blood flow (CBF) on the extent of PHE in a study of ICH patients (21). Furthermore, PHE occurs in cadaveric models of ICH in the absence of any blood flow (22). Surgical removal of ICH hematoma improves CBF on the ipsilateral side as shown in a single-photon-emission computed tomography (SPECT) study (23). Based on supportive data from animal studies, the role of surgery in ICH was explored in humans. Unfortunately, two large multicenter surgical trials in ICH patients failed to show any benefit of surgery over medical therapy (24, 25). Mean time to surgery in these trials was more than 24 h, which may have affected the possible benefit from surgery in these studies. Data from rabbit studies suggest that early surgery within 6–12 h might be of maximum benefit with lower glutamate levels, less BBB permeability, and less brain edema (26). Since large studies did not find any role for open surgery in ICH management, the role of minimally invasive surgery is currently being explored. In a rat study, recombinant tissue plasminogen activator (rt-PA) was found to be safe after ICH (27). In a phase-II ICH trial investigating hematoma evacuation using minimally invasive surgery and intra-lesional rt-PA (MISTIE-II), there was significant reduction in PHE in the minimally invasive surgical group with no ill effects of rt-PA (28). MISTIE-III trial is in underway to investigate the effect of hematoma reduction using minimally invasive surgery and intra-lesional rt-PA on long-term patient outcomes. MISTIE-III promises to be an important ICH trial and will further improve our understanding about ICH management (29). Large volume hematoma and PHE may lead to severe mass effect including uncal herniation and death. Decompressive hemicraniectomy is sometimes used in clinical practice as a life saving measure. In a retrospective case series, clot evacuation with decompressive hemicraniectomy for large ICH was associated with a favorable outcome in 29% patients (30). Decompressive hemicraniectomy trials with (31) and without (32) hematoma evacuation are currently enrolling patients to study the effect of surgery versus medical therapy in ICH patients. Although animal studies suggest reduced CBF in the perihemorrhagic region with improvement following surgery, the role of surgery still needs to be established in humans. Currently, the AHA/ASA guidelines do not recommend surgery for all ICH patients. The guidelines do recommend urgent sub-occipital craniectomy for cerebellar ICH patients with neurological decline, brainstem compression, or obstructive hydrocephalus (1).

##### Hyperosmolar Therapy

Mannitol is a commonly used osmotherapy agent for cerebral edema. The effect of mannitol on ICH-related edema and patient outcome is controversial. In rat studies, mannitol did not cause any change in the brain water content suggesting lack of its effect in ICH-related edema (33). However, a transcranial Doppler study showed that a single bolus of 100 mg mannitol in ICH patients led to higher mean flow velocity and lower pulsatility index suggesting reduced edema on the affected side (34). In two studies, mannitol was found to be safe in ICH patients, although had no clinical response (35, 36). However, Sansing et al. showed that mannitol had no effect on PHE but was related to adverse discharge outcome (37). Although one study showed transient clinical improvement with mannitol, it failed to show any improvement on computed tomography (CT) (38). A systematic review reported only two small trials about the use of mannitol in ICH and concluded that there was not enough evidence to suggest either benefit or harm with mannitol (39).

Hypertonic saline is another common mode of osmotherapy for PHE. In an ICH dog study, basal ganglia ICH was related to higher perilesional pressures, and administration of 23.4% hypertonic saline bolus led to reduction in intraparenchymal pressure; this effect was sustained for 3 h (40). An experimental dog study of ICH-induced transtentorial herniation found elevation in ICP with preservation of cerebral perfusion pressure due to concomitant arterial hypertension. Transtentorial herniation was accompanied by reduction in regional CBF in brainstem and bilateral gray/white matter. A bolus of 23.4% hypertonic saline reversed the effect of transtentorial herniation on ICP and restored CBF and cerebral oxygen consumption. The reduction in ICP after a single hypertonic saline bolus lasted 30 min (41). In a retrospective study of herniating ICH patients, two-thirds of the patients had reversal of herniation after a bolus of 30–60 ml of 23.4% hypertonic saline (42). Herniation reversal is associated with a good functional outcome in 25% patients (43). In two retrospective studies of 78 ICH patients and 132 historical controls, continuous 3% saline infusion with goal serum sodium concentration of 145–155 mmol/l and osmolality of 310–320 mOsm/kg led to less PHE on day 14, less ICP elevation, and reduced mortality with similar safety profile (cardiac arrhythmias, acute heart failure, pulmonary edema, and acute renal failure) (44). Another retrospective study also failed to show any higher risk of acute kidney injury with the use of hypertonic saline in ICH patients (45).

The effectiveness of hypertonic saline in reducing ICP and reversing herniation syndromes has been compared to the effectiveness of mannitol. In an experimental dog study with ICH in left basal ganglia, use of hypertonic saline [3% NaCl (5.3 ml/kg) or 23.4% NaCl (0.7 ml/kg)] caused reduction in ICP immediately and at 15 min. Mannitol (1 gm/kg) led to an immediate reduction in ICP, but this effect did not persist at 15 min. Only 3% NaCl caused a persistent reduction in ICP at 120 min (46). In humans, 20% ICH patients did not respond adequately (change in sodium ≤5 mEq/l) to sustained mannitol infusion (47). Although animal studies and retrospective studies suggest benefit of hyperosmolar therapy in reversing transtentorial herniation and reducing ICP, this effect needs to be proved in RCTs.

#### Targeting ICH-Related Neuroinflammation and Oxidative Stress

Numerous animal studies have investigated the effect of clot lysis and the cascade of reactions that follow. The result of this cascade is neuroinflammation and the consequent late stages of PHE (48). We present here a few animal studies and corresponding human studies, which have investigated some of the targets to reduce neuroinflammation-induced PHE. Hemoglobin released from hematoma lysis leads to iron deposition, glutamate deposition (49), free radical production, and antioxidase deactivation causing activation of matrix metalloproteinases-9 (MMP-9). MMP-9 plays an important role in BBB disruption, PHE, apoptosis, and neuronal death. Inflammation involving microglia activation and leukocyte infiltrations; and possible mitochondrial dysfunction also contribute to this oxidative stress (13). However, the role of BBB disruption in the formation of PHE has not been clearly defined in human studies. BBB disruption has been disputed as a cause of PHE by at least one group of authors (50). If BBB disruption does contribute to perihematomal edema formation, it is most likely a late effect. MMP-9 has been found to be elevated in ICH patients and is associated with HE and clinical decline (51, 52).

##### Hypothermia

Hypothermia was noted to be effective for treating brain edema and BBB disruption in animal studies. It involves upregulation of tight junction proteins and the suppression of inflammatory cytokines [interleukin 1β (IL-1β) and tumor necrosis factor α (TNF-α)] (53, 54). Hypothermia further reduces the expression of MMP-9 and apoptosis (55). In addition, elevated ambient temperature has deleterious effect on PHE. Elevated ambient temperature in patients in intensive BP reduction in acute cerebral hemorrhage trial (INTERACT) was found to be associated with worsening PHE (56) consistent with prior animal studies (57, 58). A meta-analysis of animal studies found hypothermia to be ineffective in reducing brain edema (48) contrary to a human study of ICH patients (12 experimental versus 25 control) where hypothermia up to 35°C showed more reduction in edema in the experimental group (59). A phase-II hypothermia trial in ICH is exploring role of hypothermia (32–34°C) in improving edema and patient outcomes (60). Although there is conflicting data in the animal studies, the role of hypothermia in reducing the PHE or improving patient outcomes warrants future studies.

##### Iron Chelators

Hemoglobin degradation leading to iron deposition contributes to PHE. In animal models, heme diffused rapidly through perivascular spaces and induced heme oxygenase-1 enzyme that led to accumulation of ferritin and hemosiderin; generation of free radicals; neuronal injury; and BBB disruption (61). In a rat study, ferrous iron was found to cause brain injury, making this a potential target for chelation therapy (62). Lipocalin-2, a siderophore-binding protein, present in astrocytes, microglia, neurons, and endothelial cells, is involved in cellular iron transport and neuroinflammation. Lipocalin-2 plays an active role in iron toxicity after ICH with ferritin upregulation in ipsilateral basal ganglia, microglial activation, more brain swelling, brain atrophy, and neurological deficits (63). In ICH rat studies, brain pathology showed iron overload and upregulation of iron-handling proteins (transferrin, transferrin receptor, and ferritin) (64). In human studies of ICH patients, high serum ferritin (65–67), low serum iron, and low transferrin were correlated with high hematoma volume, early PHE, and poor outcome at 90 days (68). Another study suggested that the effect of ferritin on poor clinical outcomes is not a direct cause and effect but a marker of iron-related PHE, which in turn causes a direct effect on patient outcomes (69). In an MRI study in ICH patients, increased iron deposition in the hematoma and the contralateral globus pallidus was associated with increased PHE suggesting iron overload in the brain may contribute to PHE (70).

In rat and piglet studies, deferoxamine, an iron chelator, reduced ferritin upregulation, hematoma lysis, hematoma cavity size, neuronal death, and neurological deficits (71–74). The iron chelator enters the perifocal reactive zone *via* the disrupted BBB (61). A phase-I study of 20 patients found deferoxamine (7–62 mg/kg/day) to be safe in ICH patients (75). A controlled study of 42 patients showed slowing of hematoma absorption and reduction in PHE using deferoxamine without any clinical benefit (76). Currently, two controlled trials are ongoing to study the effect of deferoxamine on PHE and long-term clinical outcomes (77, 78).

Minocycline is another iron chelator, MMP-9 inhibitor, and anti-inflammatory drug, which has reduced iron-related neuronal injury in animals along with reducing PHE, neuronal death, improving BBB, and improving neurobehavioral outcomes (79–83). A combined phase-I and phase-II study is currently recruiting 24 ICH patients to test the safety and pharmacokinetics of minocycline and to simultaneously test its effect on blood biomarkers (84). Animal and early human studies support a toxic role of iron in ICH-related neuroinflammation and oxidative damage. Ongoing clinical trials will explore the role of iron chelation on PHE and patient outcomes.

##### Statins

Statins are inhibitors of 3-hydroxy-3-methyl-glutaryl-coenzyme-A (HMG-CoA) reductase and are commonly used medications for dyslipidemia. In animal studies, statins showed less neuronal tissue loss and inflammation; increased cell proliferation, angiogenesis, and synaptogenesis in the perihemorrhagic zone with better functional outcomes (85–87). Statins’ neuroprotective actions may be explained by their ability to induce expression of vascular endothelial growth factor (VEGF), brain-derived neurotrophic factor (BDNF), and nerve growth factor (NGF) (88) to reduce activated microglia, MMP-9 (89), and cytokine-mediated inflammation (less TNF-α and higher interleukin-10 levels) (90, 91). Statins also reduce BBB dysfunction, edema, tissue loss, and improve CBF in the perihemorrhagic zone (92, 93). Combination of granulocyte-colony stimulating factor (G-CSF) and statins further enhance their neuroprotective capacity with less cell apoptosis than with either agents alone (94). Combination of statins and deferoxamine showed a greater effect on PHE and hematoma volume than either agent alone (95). Unlike animal studies, statin use in humans has been linked to increased PHE volume in ICH patients with no effect on CBF (96). One study of 303 ICH patients with 71 statin users showed higher initial and final hematoma volume in statin users (97). Conversely, a pilot study of 18 patients showed a possible protective role of rosuvastatin in ICH patients with lower mortality; however, after adjustment for disease severity, the hazards ratio was not significant (HR 0.20; 95% CI 0.02–1.67) (98). A meta-analysis of 17 studies with 3455 ICH patients exposed to statins showed a significant risk reduction of mortality (OR 0.73; 95% CI 0.54–0.97) (99). Three studies assessed the effect of statin discontinuation following ICH and noted a trend toward reduction in mortality risk with continuation of statins (OR 0.14; 95% CI 0.1–0.20) (99–102). Animal studies suggesting a beneficial role of statins in reducing neuroinflammation and retrospective human studies showing conflicting results set the stage for a large phase-III trial to explore the potential benefit of continuing statins in ICH patients who were receiving statins prior to ICH onset.

##### Memantine

Animal studies demonstrate that elevated glutamate after ICH contributes to BBB breakdown, neuronal death, and brain edema *via* stimulating *N*-methyl-d-aspartate (NMDA) receptors (49). A meta-analysis of animal studies did not show any benefit of glutamate receptor antagonists (Amantadine, Memantine, and YM872) on PHE (48). However, the effect of memantine has been consistently positive. Memantine, an FDA-approved NMDA antagonist for Alzheimer’s disease, showed benefit for ICH in animal studies with reduced hematoma growth, reduced inflammation with low MMP-9 level, less apoptosis, and better functional recovery (103). A similar effect was seen along with reduced PHE in another study with the combination of memantine and celecoxib, a selective cyclooxygenase-2 (COX-2) inhibitor. The effect of this combination on PHE was better than the use of single agent (104). A single-centered RCT of 64 ICH patients confirmed the beneficial effect of memantine with better modified Rankin score and Barthel index at 90 days (105). With supportive data from animal studies and a single-centered RCT in humans, there is enough preliminary data to support a large multicenter RCT to confirm these findings. There are currently no registered trials looking at role of memantine in ICH.

##### Celecoxib

Animal models with ICH have shown that celecoxib, a selective cyclooxygenase-2 (COX-2) inhibitor, has anti-inflammatory, antioxidant, and neuroprotective effects. Celecoxib use was associated with reduced brain edema, prostaglandin E2 level, perihematomal cell death, and better neurobehavioral recovery (106). As mentioned earlier, celecoxib in combination with memantine was associated with less apoptosis, less inflammation, reduced hematoma volume and brain edema, and better functional recovery. The effect was greater than each drug alone (104). In a retrospective study of ICH patients, celecoxib (400 mg/day) for ≥7 days significantly reduced the hematoma volume and PHE as compared to controls, without any difference in adverse events (107). These findings were further confirmed in multicenter RCT of 44 ICH patients where celecoxib 400 mg twice a day was given for 14 days (108). To confirm the findings of animal studies and a single small RCT, a larger trial is needed to show the effect of celecoxib on clinical outcomes in ICH patients.

##### Fingolimod

Animal studies showed that PHE results from inflammatory response with perilesional infiltration of neutrophils and macrophages, activation of microglia, ICAM-1 immunoreactivity, and leakage of BBB (109, 110). A sphingosine-1-phosphate receptor (S1PR) modulator, fingolimod was found to have mixed results in animal studies varying from no effect on PHE or clinical outcomes (111) to reduced edema with better neurobehavioral recovery (112–114). The positive effect from fingolimod is due to reduction in lymphocytes, markers of inflammation [intercellular adhesion molecule-1 (ICAM-1), interferon-γ, and interleukin-17], apoptosis, and brain atrophy (112–114). In a human study of 23 patients, fingolimod use was associated with less inflammation (reduced ICAM-1, lymphocytes, natural killer cells, and MMP-9 levels) and positive effect on BBB (115). In a phase-II study, fingolimod use within 72 h of ICH onset was associated with less PHE and improvement in clinical outcomes at 3 months without any increase in side effects (116). Fingolimod use is limited due to its potential for cardiotoxicity. RP101075, a selective S1PR1 agonist, has less potential for cardiotoxicity, yet, it retains the ability to reduce cerebral edema in mice. RP101075 results in less cytokine expression, higher BBB integrity, less infiltration of lymphocytes, neutrophils, and microglia, and less cell death (117). Human studies with RP101075 are awaited. Although animal studies and a phase-II human study suggest a possible beneficial role of fingolimod, a phase-III study is needed to confirm these findings.

##### Hyperglycemia

A rat study has shown that induced hyperglycemia leads to more cell death in perihemorrhagic region and more global edema (118). Another animal study found a correlation between perilesional hyperglycemia and PHE and suggested a possible role for hyperglycemia in BBB breakdown (119). A *post hoc* exploratory analysis of the antihypertensive treatment of acute cerebral hemorrhage (ATACH) trial found a trend toward worsening of PHE with hyperglycemia, but it was not statistically significant (relative risk 1.25; CI 0.73–2.13) (120). Another retrospective study did not find any relationship between hyperglycemia and PHE in ICH patients (121). Supporting data from animal studies and conflicting results from two small human studies point toward need for more data to understand role of hyperglycemia on PHE in ICH patients before future trials can be planned. AHA/ASA guidelines recommend avoiding hypoglycemia or hyperglycemia in ICH patients (1).

Current AHA/ASA guidelines do not recommend routine use of any anti-inflammatory agents in ICH patients (1).

## Hematoma Expansion

About one-third of the ICH patients have HE within 3 h and one-sixth in the first 6 h causing early neurological decline and increasing the risk of long-term poor outcomes (1, 122). HE may be related to prior antithrombotic use, computed tomography angiography (CTA) spot sign (123), heterogeneous hematoma density, irregular hematoma shape (124), platelet dysfunction, and higher BP (1). Currently, there are no approved therapies for reducing HE. We will discuss some potential methods of reducing HE for which we have basic science research, or clinical evidence, regarding their potential application at the bedside.

### Treatment of Hematoma Expansion

#### Activated Recombinant Factor VII

An animal study found hematoma reduction in experimental ICH rats with use of activated recombinant factor VII (rFVIIa) (125). In humans, an open-label uncontrolled study found benefit of rFVIIa in controlling ICH in patients with hemophilia A or B (126). The benefit of rFVIIa in hemophilia triggered interest for its use in ICH patients without hemophilia and a phase-II safety study of 48 patients found it to be safe with adverse events (rash, vomiting, fever, ECG T-wave inversion, and deep venous thrombosis) in 12.5% patients (122). This safety study led to a RCT of 399 patients, which found reduction in hematoma volume with rFVIIa as compared to placebo (11–16 versus 29%). Hematoma growth was reduced by 3–6 ml depending on the rFVIIa dose (40–160 μg/kg). Poor outcome including mortality was reduced at 90 days, despite rFVIIa-related serious adverse events in 2.3% patients (myocardial or cerebral infarction) (127). A follow-up efficacy study of 841 patients again showed reduction in HE with rFVIIa as compared to placebo (11–18 versus 26%). However, in this larger study, there was an excess in arterial thrombotic events and there was no overall clinical benefit, while the trend was toward greater death and disability in the treatment group with rFVIIa as compared to placebo (26–29 versus 24%) (128).

Few studies have explored the potential benefit of rFVIIa during the perioperative management for ICH patients. Use of rFVIIa (40–90 μg/kg) prior to hematoma evacuation in 15 patients with mean preoperative ICH volume of 60 ml was found to be safe with good outcome in 73% patients (129). In another placebo-controlled open-label study, rFVIIa was used during hematoma evacuation (100 μg/kg) with no difference in postoperative hematoma volume and adverse events (130). It remains to be seen how rFVIIa would find a place in ICH management knowing that animal and phase-III studies have shown that it reduces HE. The clinical benefit would have to outweigh the side effect of arterial thrombosis and the right dose and clinical setting would need to be narrowly defined. Currently, AHA/ASA and neurocritical care society (NCS) guidelines recommend against use of rFVIIa in ICH patients (1, 131).

#### Warfarin Reversal

Oral anticoagulation (OAC) is a common risk factor for ICH associated with higher rate of HE and poor clinical outcomes. In a rat study, higher INR was associated with higher hematoma volume. OAC-related hematoma enlargement occurred in the first 3 h of ICH onset (132). Interestingly, warfarin also reduced the cell death and MMP-9 levels; however, HE was out of proportion than this positive effect (133). Warfarin-related HE has been confirmed in several human studies (134–137). Following warfarin-related ICH, warfarin is held and emergent reversal of the warfarin effect is needed. A meta-analysis comparing agents: prothrombin complex concentrate (PCC) and fresh frozen plasma (FFP) for warfarin reversal included five RCTs and eight observational studies. PCC use was associated with significant normalization of INR, shorter time to INR correction, lower risk of posttransfusion volume overload, and less mortality without any added risk of thromboembolism (138). A RCT of PCC versus FFP in ICH patients is currently recruiting patients (139). Supporting animal studies and phase-III studies have led to NCS recommendations of administering PCC with intravenous vitamin K over FFP and vitamin K combination for warfarin reversal for ICH patients (131).

#### Newer Anticoagulants Reversal

In large RCTs of anticoagulation after atrial fibrillation, direct thrombin inhibitors (dabigatran) and direct factor Xa inhibitors (apixaban, rivaroxaban) were associated with lower annual rate of hemorrhagic stroke when compared to warfarin (140), which made them the agents of choice for secondary stroke prevention in atrial fibrillation patients. Their main limiting factor was non-availability of a reversal agent in case of hemorrhage. There was uncertainty around the effect of these medications on ICH volume. An animal study using dabigatran etexilate did not find any significant change in ICH volume when compared to placebo (141). In humans, most cases of newer anticoagulants (NOAC)-related ICH has reported hematoma size less than 30 cc with very low risk for HE (136, 142–144). Need for reversal agents for these agents led to research on Idarucizumab, an antibody fragment, which is currently food and drug administration (FDA) approved. It was studied in 90 patients (51 patients with acute serious bleeding) taking dabigatran and was found to normalize the abnormal thrombin and ecarin clotting time in more than 80% patients within minutes resulting in normal hemostasis within 11.4 h. There were 5.6% patients with thrombotic events over the next 30 days (145). In a rat study, Idarucizumab was associated with reduced mortality (146). Andexanet alfa, a recombinant modified human factor Xa decoy protein, was evaluated in 67 patients with acute major gastrointestinal or intracranial bleeding while on factor Xa inhibitor. Anti-factor Xa activity decreased consistently for 2 h by more than 80% from baseline. Clinical hemostasis was achieved in 79% patients within 12 h; however, there were 18% patients with thrombotic events during the 30-day follow-up (147). Andexanet alfa is not yet approved by FDA. Future trials may help us understand if this change in hemostasis using these novel medications will translate into better clinical outcome.

Another important issue in ICH patients requiring anticoagulation for atrial fibrillation or mechanical heart valve is the timing of resumption of anticoagulation after ICH. There is no previous RCT answering this question and a phase-II study is currently recruiting patients to compare the safety of apixaban versus antiplatelet agents or no antiplatelet/anticoagulation agent in atrial fibrillation patients after ICH (148). Current NCS guidelines recommend using activated charcoal and PCC if needed for direct factor Xa inhibitor-related ICH. They recommend use of Idarucizumab for dabigatran-related ICH, which is supported by animal and current human studies (131).

#### Antiplatelet Reversal

Elderly patients with ICH are often taking antiplatelet agents prior to admission. An experimental rat study did not find any significant change in hematoma volume or neurological deficits between rats that were pretreated with antiplatelet agent before ICH versus those who were not pretreated (149). However, in humans, prior antiplatelet agent use has been associated with higher risk of HE and poor outcome, with aspirin having the lowest risk as compared to clopidogrel or ticlopidine (135, 150). In a pilot study of 14 ICH patients, desmopressin use was found to be safe and resulted in improvement of platelet function warranting larger studies to confirm these findings (151). A recently completed European randomized, open-label, and phase-III trial studying platelet transfusion versus standard care after acute ICH with prior antiplatelet therapy (PATCH) found two times higher odds of death or dependence at 3 months in patients receiving platelet transfusion (152). It remains to be seen if findings from the PATCH trial will be validated in another blinded RCT in a continent other than Europe. A larger RCT is awaited to study the efficacy of desmopressin on ICH patient outcomes. Despite conflicting data from the animal study and early human studies, current NCS guidelines recommend using a dose of desmopressin (0.4 mcg/kg IV) in ICH patients on antiplatelet agents prior to admission and platelet transfusion only in patients scheduled to undergo neurosurgery (131).

#### BP Reduction

Hypertension is common in ICH patients with two-thirds of patients having systolic BP more than 140 mm Hg at presentation (153). A rat study found acute BP elevation in either normotensive or hypertensive rats after ICH, which is associated with higher HE, more brain swelling, and worse neurological deficits (154, 155). Since acute ICH leads to activation of the adrenergic system, the role of antiadrenergic agents needs to be explored. There are conflicting data about the effect of beta-blockade on clinical outcomes following ICH. Inpatient use of antiadrenergic medications (beta-blockers or clonidine) in 303 patients was associated with less PHE and better clinical outcome after controlling for hematoma volume and BP (156). This protective effect of antiadrenergic medications was confirmed by another study of 138 ICH patients where in-hospital atenolol use was associated with less mortality, systemic inflammatory response, and pneumonia (157). This protective effect of antiadrenergic medications was challenged by a large cohort study from Denmark with 11,779 ICH patients who showed no benefit of preadmission beta blockers over long-term mortality (158). A US study of 426 ICH patients did not find any class-specific benefit of beta-blockers over other antihypertensives (159).

Another area of interest in ICH is intense BP control defined as less than 140/90 mm Hg. ATACH-II, a recent RCT studying role of intensive BP control versus standard BP management in ICH patients found a trend toward less HE (≥33%) in the intensive BP arm (18.9 versus 24.4%, *p* = 0.09) (160). Another similar trial also showed a trend toward less absolute hematoma volume change in the intensive arm as compared to standard BP management (3.1 versus 4.9 ml, *p* = 0.09) (161). However, both these trials failed to show any benefit in clinical outcomes with intensive BP control. A pooled analysis of five RCTs also showed no benefit of aggressive BP lowering on patient outcomes or HE (162). A new open-label, dose-escalating study of glyceryl trinitrate transdermal patch in ICH patients is underway, to study the effect of BP control in the first 2 h of symptom onset by starting medication *via* the emergency medical services (163). Although animal studies suggested possible role of BP control in reducing HE in ICH patients, failed phase-III RCTs do not support it. There is probably some role of BP control on HE; however, the right BP target and timing to achieve it needs further evaluation. AHA/ASA guidelines suggest that acute systolic BP lowering to 140 mm Hg is safe and can be effective for improving functional outcomes (1). It remains to be seen if these guidelines would change after the recently published ATACH II trial.

#### Tranexamic Acid

Tranexamic acid is an antifibrinolytic agent, which acts by inhibiting conversion of plasminogen to plasmin. In an experimental rat ICH study, combination of plasminogen and thrombin was noted to cause significant neuronal damage, which was prevented by tranexamic acid (164). A RCT including 30 ICH patients found that tranexamic acid group had no significant hematoma growth as compared to control (165). Another pilot study showed that two patients per month can be enrolled to study the effect of tranexamic acid on ICH patients (166). Animal studies and positive pilot studies support the ongoing two phase-II studies to explore the benefit of tranexamic acid for ICH patients (167, 168). Lack of strong evidence explains why tranexamic acid is missing in the current ICH guidelines from AHA/ASA (1).

#### Memantine

In addition to its possible benefit in reducing PHE, memantine has been purported to have some role in preventing HE. In a rat study, memantine use was associated with reduction in tissue plasminogen activator (tPA), urokinase plasminogen activator (uPA), MMP-9, and HE (103). In a study of 186 ICH patients, elevated MMP-9 was independently associated with the risk of HE (OR 15.65, 95% CI: 5.30–46.15) (52). Another rat study with a combination therapy with memantine and celecoxib showed a reduction in cerebral inflammation, apoptosis, hematoma volume, and better functional recovery (104). A single-centered RCT of 64 ICH patients confirmed the beneficial effect to memantine in humans on outcomes, but they did not study its effect on HE (105). The effect of memantine on HE and clinical outcomes in animals would need further evidence in humans to achieve clinical utility. There are currently no registered trials looking at the role of memantine in ICH; memantine is not listed in the current AHA/ASA guidelines (1).

#### Hyperglycemia

A rat study found that hyperglycemia *via* its interaction with plasma kallikrein, leads to HE *via* inhibition of collagen-induced platelet aggregation (169). Another animal study found protective role of 17-beta estradiol in preventing hyperglycemia-induced harmful effect on HE. A *post hoc* exploratory analysis of ATACH trial found a statistically significant worsening of HE with hyperglycemia (120). Hyperglycemia associated HE worsening was also seen in another retrospective study, but the association was not statistically significant (121). Prospective cohort studies are needed to understand the association between hypoglycemia and HE. As mentioned previously, AHA/ASA guidelines recommend avoiding hypoglycemia or hyperglycemia in ICH patients (1).

## Conclusion

Despite extensive animal data supporting numerous therapeutic targets to prevent morbidity and mortality in ICH, very few studies have made the journey from bench to bedside. Failures of recent ICH trials have given us an opportunity to rethink the paradigms of the management for ICH patients. What are the critical steps in improving the outcomes of these patients? Is early hematoma evacuation using minimally invasive surgery crucial to minimizing injury by preventing the cascade of secondary injury? Is the need for critical care (reversal of antiplatelet agents and anticoagulation agents, BP control, hyperglycemia control, cerebral edema management, venous thromboembolism prevention) the key to prevention of secondary injury? Is it the use of anti-inflammatory agents like statins and Fingolimod? Or is there some other, yet undetermined, factor? An MRI animal study suggests that mortality may be determined less by hematoma volume than by the invasion of internal capsule suggesting an anatomically more eloquent basis for clinical deterioration (170). Patient selection may be the key as not everyone would benefit from either surgery or medical therapy, possibly depending on damage to an eloquent area or pathway during the primary injury.

## Author Contributions

MM: first draft preparation, literature review, critical review of manuscript, and revision after peer review. AL: second draft preparation, literature review, and critical review of manuscript.

## Conflict of Interest Statement

The authors declare that the research was conducted in the absence of any commercial or financial relationships that could be construed as a potential conflict of interest.

## References

[1] Hemphill JC III Greenberg SM Anderson CS Becker K Bendok BR Cushman M Guidelines for the management of spontaneous intracerebral hemorrhage: a guideline for healthcare professionals from the American Heart Association/American Stroke Association. Stroke (2015) 46:2032–60. 10.1161/STR.0000000000000069 26022637

[2] Mutlu N Berry RG Alpers BJ Massive cerebral hemorrhage. Clinical and pathological correlations. Arch Neurol (1963) 8:644–61. 10.1001/archneur.1963.00460060074008 14065777

[3] Wagner KR Xi G Hua Y Kleinholz M de Courten-Myers GM Myers RE Lobar intracerebral hemorrhage model in pigs: rapid edema development in perihematomal white matter. Stroke (1996) 27:490–7. 10.1161/01.STR.27.3.490 8610319

[4] Arima H Wang JG Huang Y Heeley E Skulina C Parsons MW Significance of perihematomal edema in acute intracerebral hemorrhage: the INTERACT trial. Neurology (2009) 73:1963–8. 10.1212/WNL.0b013e3181c55ed3 19996072 PMC2796457

[5] Tomita H Ito U Ohno K Hirakawa K. Chronological changes in brain edema induced by experimental intracerebral hematoma in cats. Acta Neurochir Suppl (Wien) (1994) 60:558–60. 7976649 10.1007/978-3-7091-9334-1_154

[6] Venkatasubramanian C Mlynash M Finley-Caulfield A Eyngorn I Kalimuthu R Snider RW Natural history of perihematomal edema after intracerebral hemorrhage measured by serial magnetic resonance imaging. Stroke (2011) 42:73–80. 10.1161/STROKEAHA.110.590646 21164136 PMC3074599

[7] Gebel JM Jr Jauch EC Brott TG Khoury J Sauerbeck L Salisbury S Relative edema volume is a predictor of outcome in patients with hyperacute spontaneous intracerebral hemorrhage. Stroke (2002) 33:2636–41. 10.1161/01.STR.0000035284.12699.84 12411654

[8] Yang J Arima H Wu G Heeley E Delcourt C Zhou J Prognostic significance of perihematomal edema in acute intracerebral hemorrhage: pooled analysis from the intensive blood pressure reduction in acute cerebral hemorrhage trial studies. Stroke (2015) 46:1009–13. 10.1161/STROKEAHA.114.007154 25712944

[9] Appelboom G Bruce SS Hickman ZL Zacharia BE Carpenter AM Vaughan KA Volume-dependent effect of perihaematomal oedema on outcome for spontaneous intracerebral haemorrhages. J Neurol Neurosurg Psychiatry (2013) 84:488–93. 10.1136/jnnp-2012-303160 23345281

[10] Murthy SB Moradiya Y Dawson J Lees KR Hanley DF Ziai WC Perihematomal edema and functional outcomes in intracerebral hemorrhage: influence of hematoma volume and location. Stroke (2015) 46:3088–92. 10.1161/STROKEAHA.115.010054 26396030

[11] Qureshi AI Majidi S Gilani WI Palesch YY Martin R Novitzke J Increased brain volume among good grade patients with intracerebral hemorrhage. Results from the antihypertensive treatment of acute cerebral hemorrhage (ATACH) study. Neurocrit Care (2014) 20:470–5. 10.1007/s12028-013-9842-1 23609118

[12] Butcher KS Baird T MacGregor L Desmond P Tress B Davis S. Perihematomal edema in primary intracerebral hemorrhage is plasma derived. Stroke (2004) 35:1879–85. 10.1161/01.STR.0000131807.54742.1a 15178826

[13] Hu X Tao C Gan Q Zheng J Li H You C. Oxidative stress in intracerebral hemorrhage: sources, mechanisms, and therapeutic targets. Oxid Med Cell Longev (2016) 2016:3215391. 10.1155/2016/3215391 26843907 PMC4710930

[14] Lee KR Colon GP Betz AL Keep RF Kim S Hoff JT. Edema from intracerebral hemorrhage: the role of thrombin. J Neurosurg (1996) 84:91–6. 10.3171/jns.1996.84.1.0091 8613842

[15] Xi G Hua Y Bhasin RR Ennis SR Keep RF Hoff JT. Mechanisms of edema formation after intracerebral hemorrhage: effects of extravasated red blood cells on blood flow and blood-brain barrier integrity. Stroke (2001) 32:2932–8. 10.1161/hs1201.099820 11739998

[16] Xi G Keep RF Hoff JT. Mechanisms of brain injury after intracerebral haemorrhage. Lancet Neurol (2006) 5:53–63. 10.1016/S1474-4422(05)70283-0 16361023

[17] Lyden PD Shuaib A Lees KR Davalos A Davis SM Diener HC Safety and tolerability of NXY-059 for acute intracerebral hemorrhage: the CHANT trial. Stroke (2007) 38:2262–9. 10.1161/STROKEAHA.106.472746 17569876

[18] Orakcioglu B Becker K Sakowitz OW Herweh C Köhrmann M Huttner HB MRI of the perihemorrhagic zone in a rat ICH model: effect of hematoma evacuation. Neurocrit Care (2008) 8:448–55. 10.1007/s12028-007-9047-6 18188706

[19] Orakcioglu B Fiebach JB Steiner T Kollmar R Jüttler E Becker K Evolution of early perihemorrhagic changes – ischemia vs. edema: an MRI study in rats. Exp Neurol (2005) 193:369–76. 10.1016/j.expneurol.2005.01.017 15869939

[20] Orakcioglu B Becker K Sakowitz OW Unterberg A Schellinger PD. Serial diffusion and perfusion MRI analysis of the perihemorrhagic zone in a rat ICH model. Acta Neurochir Suppl (2008) 103:15–8. 10.1007/978-3-211-76589-0_5 18496940

[21] McCourt R Gould B Gioia L Kate M Coutts SB Dowlatshahi D Cerebral perfusion and blood pressure do not affect perihematoma edema growth in acute intracerebral hemorrhage. Stroke (2014) 45:1292–8. 10.1161/STROKEAHA.113.003194 24692481

[22] Majidi S Rahim B Gilani SI Gilani WI Adil MM Qureshi AI. CT evolution of hematoma and surrounding hypodensity in a cadaveric model of intracerebral hemorrhage. J Neuroimaging (2016) 26:346–50. 10.1111/jon.12306 26459244

[23] Siddique MS Fernandes HM Arene NU Wooldridge TD Fenwick JD Mendelow AD. Changes in cerebral blood flow as measured by HMPAO SPECT in patients following spontaneous intracerebral haemorrhage. Acta Neurochir Suppl (2000) 76:517–20. 11450081 10.1007/978-3-7091-6346-7_108

[24] Mendelow AD Gregson BA Fernandes HM Murray GD Teasdale GM Hope DT Early surgery versus initial conservative treatment in patients with spontaneous supratentorial intracerebral haematomas in the international surgical trial in intracerebral haemorrhage (STICH): a randomised trial. Lancet (2005) 365:387–97. 10.1016/S0140-6736(05)70233-6 15680453

[25] Mendelow AD Gregson BA Rowan EN Murray GD Gholkar A Mitchell PM Early surgery versus initial conservative treatment in patients with spontaneous supratentorial lobar intracerebral haematomas (STICH II): a randomised trial. Lancet (2013) 382:397–408. 10.1016/S0140-6736(13)60986-1 23726393 PMC3906609

[26] Wu G Wang L Wang F Feng A Sheng F. Minimally invasive procedures for intracerebral hematoma evacuation in early stages decrease perihematomal glutamate level and improve neurological function in a rabbit model of ICH. Brain Res (2013) 1492:140–7. 10.1016/j.brainres.2012.11.023 23183043

[27] Pfeilschifter W Kashefiolasl S Lauer A Steinmetz H Foerch C Hematoma expansion in experimental intracerebral hemorrhage is not altered by peracute treatment with recombinant tissue plasminogen activator. Neuroscience (2013) 250:181–8. 10.1016/j.neuroscience.2013.07.003 23856067

[28] Mould WA Carhuapoma JR Muschelli J Lane K Morgan TC McBee NA Minimally invasive surgery plus recombinant tissue-type plasminogen activator for intracerebral hemorrhage evacuation decreases perihematomal edema. Stroke (2013) 44:627–34. 10.1161/STROKEAHA.111.000411 23391763 PMC4124642

[29] National Institute of Neurological Disorders and Stroke, Johns Hopkins University. Minimally Invasive Surgery Plus Rt-PA for ICH Evacuation Phase III (MISTIE III). Bethesda, MD: National Library of Medicine (US) (2000). Available from: https://clinicaltrials.gov/show/NCT01827046

[30] Esquenazi Y Savitz SI El Khoury R McIntosh MA Grotta JC Tandon N. Decompressive hemicraniectomy with or without clot evacuation for large spontaneous supratentorial intracerebral hemorrhages. Clin Neurol Neurosurg (2015) 128:117–22. 10.1016/j.clineuro.2014.11.015 25496934

[31] Southwest Hospital, China. Decompressive Craniectomy Combined with Hematoma Removal to Treat ICH (CARICH). Bethesda, MD: National Library of Medicine (US) (2000). Available from: https://clinicaltrials.gov/show/NCT02135783

[32] University Hospital Inselspital, Berne. Decompressive Hemicraniectomy in Intracerebral Hemorrhage (SWITCH). Bethesda, MD: National Library of Medicine (US) (2000). Available from: https://clinicaltrials.gov/show/NCT02258919

[33] Rosenberg GA Estrada EY. Atrial natriuretic peptide blocks hemorrhagic brain edema after 4-hour delay in rats. Stroke (1995) 26:874–7. 10.1161/01.STR.26.5.874 7740582

[34] Vicenzini E Ricciardi MC Zuco C Sirimarco G Di Piero V Lenzi GL. Effects of a single mannitol bolus on cerebral hemodynamics in intracerebral hemorrhage: a transcranial Doppler study. Cerebrovasc Dis (2011) 32:447–53. 10.1159/000330639 22005320

[35] Wang X Arima H Yang J Zhang S Wu G Woodward M Mannitol and outcome in intracerebral hemorrhage: propensity score and multivariable intensive blood pressure reduction in acute cerebral hemorrhage trial 2 results. Stroke (2015) 46:2762–7. 10.1161/STROKEAHA.115.009357 26265125

[36] Misra UK Kalita J Ranjan P Mandal SK. Mannitol in intracerebral hemorrhage: a randomized controlled study. J Neurol Sci (2005) 234:41–5. 10.1016/j.jns.2005.03.038 15936036

[37] Sansing LH Kaznatcheeva EA Perkins CJ Komaroff E Gutman FB Newman GC. Edema after intracerebral hemorrhage: correlations with coagulation parameters and treatment. J Neurosurg (2003) 98:985–92. 10.3171/jns.2003.98.5.0985 12744358

[38] Misra UK Kalita J Vajpayee A Phadke RV Hadique A Savlani V. Effect of single mannitol bolus in intracerebral hemorrhage. Eur J Neurol (2007) 14:1118–23. 10.1111/j.1468-1331.2007.01918.x 17727664

[39] Bereczki D Fekete I Prado GF Liu M Mannitol for acute stroke. Cochrane Database Syst Rev (2007) 3:Cd001153. 10.1002/14651858.CD001153.pub2 PMC7032636 17636655

[40] Qureshi AI Suri MF Ringer AJ Guterman LR Hopkins LN. Regional intraparenchymal pressure differences in experimental intracerebral hemorrhage: effect of hypertonic saline. Crit Care Med (2002) 30:435–41. 10.1097/00003246-200202000-00028 11889326

[41] Qureshi AI Wilson DA Traystman RJ. Treatment of transtentorial herniation unresponsive to hyperventilation using hypertonic saline in dogs: effect on cerebral blood flow and metabolism. J Neurosurg Anesthesiol (2002) 14:22–30. 10.1097/00008506-200201000-00005 11773819

[42] Koenig MA Bryan M Lewin JL III Mirski MA Geocadin RG Stevens RD. Reversal of transtentorial herniation with hypertonic saline. Neurology (2008) 70:1023–9. 10.1212/01.wnl.0000304042.05557.60 18272864

[43] Qureshi AI Geocadin RG Suarez JI Ulatowski JA. Long-term outcome after medical reversal of transtentorial herniation in patients with supratentorial mass lesions. Crit Care Med (2000) 28:1556–64. 10.1097/00003246-200005000-00049 10834711

[44] Wagner I Hauer EM Staykov D Volbers B Dörfler A Schwab S Effects of continuous hypertonic saline infusion on perihemorrhagic edema evolution. Stroke (2011) 42:1540–5. 10.1161/STROKEAHA.110.609479 21512173

[45] Corry JJ Varelas P Abdelhak T Morris S Hawley M Hawkins A Variable change in renal function by hypertonic saline. World J Crit Care Med (2014) 3:61–7. 10.5492/wjccm.v3.i2.61 24892021 PMC4038814

[46] Qureshi AI Wilson DA Traystman RJ. Treatment of elevated intracranial pressure in experimental intracerebral hemorrhage: comparison between mannitol and hypertonic saline. Neurosurgery (1999) 44:1055–63; discussion 63–4. 10.1097/00006123-199905000-00064 10232539

[47] Keyrouz SG Dhar R Diringer MN. Variation in osmotic response to sustained mannitol administration. Neurocrit Care (2008) 9:204–9. 10.1007/s12028-008-9118-3 18563637 PMC2575015

[48] Frantzias J Sena ES Macleod MR Al-Shahi Salman R. Treatment of intracerebral hemorrhage in animal models: meta-analysis. Ann Neurol (2011) 69:389–99. 10.1002/ana.22243 21387381

[49] Wu G Sun S Sheng F Wang L Wang F. Perihematomal glutamate level is associated with the blood-brain barrier disruption in a rabbit model of intracerebral hemorrhage. Springerplus (2013) 2:358. 10.1186/2193-1801-2-358 23961420 PMC3738910

[50] McCourt R Gould B Kate M Asdaghi N Kosior JC Coutts S Blood-brain barrier compromise does not predict perihematoma edema growth in intracerebral hemorrhage. Stroke (2015) 46:954–60. 10.1161/STROKEAHA.114.007544 25700288

[51] Petrovska-Cvetkovska D Dolnenec-Baneva N Nikodijevik D Chepreganova-Changovska T. Correlative study between serum matrix metalloproteinase-9 values and neurologic deficit in acute, primary, supratentorial, intracerebral haemorrhage. Pril (Makedon Akad Nauk Umet Odd Med Nauki) (2014) 35:39–44. 25500674 10.2478/prilozi-2014-0005

[52] Yang Q Zhuang X Peng F Zheng W Relationship of plasma matrix metalloproteinase-9 and hematoma expansion in acute hypertensive cerebral hemorrhage. Int J Neurosci (2016) 126:213–8. 26708160 10.3109/00207454.2015.1007372

[53] Fingas M Clark DL Colbourne F. The effects of selective brain hypothermia on intracerebral hemorrhage in rats. Exp Neurol (2007) 208:277–84. 10.1016/j.expneurol.2007.08.018 17927984

[54] Sun H Tang Y Guan X Li L Wang D. Effects of selective hypothermia on blood-brain barrier integrity and tight junction protein expression levels after intracerebral hemorrhage in rats. Biol Chem (2013) 394:1317–24. 10.1515/hsz-2013-0142 23828426

[55] Sun H Tang Y Li L Guan X Wang D. Effects of local hypothermia on neuronal cell apoptosis after intracerebral hemorrhage in rats. J Nutr Health Aging (2015) 19:291–8. 10.1007/s12603-015-0469-0 25732214 PMC12877534

[56] Zheng D Arima H Heeley E Karpin A Yang J Chalmers J Ambient temperature and volume of perihematomal edema in acute intracerebral haemorrhage: the INTERACT1 study. Int J Stroke (2015) 10:25–7. 10.1111/ijs.12389 25345354

[57] Muresanu DF Sharma A Sharma HS. Diabetes aggravates heat stress-induced blood-brain barrier breakdown, reduction in cerebral blood flow, edema formation, and brain pathology: possible neuroprotection with growth hormone. Ann N Y Acad Sci (2010) 1199:15–26. 10.1111/j.1749-6632.2009.05328.x 20633105

[58] Lin LS Chiu WT Shih CJ Lin MT. Influence of thermal stress and various agents on the brain edema formation in rats following a cryogenic brain lesion. Chin J Physiol (1989) 32:41–7. 2517859

[59] Kollmar R Staykov D Dorfler A Schellinger PD Schwab S Bardutzky J. Hypothermia reduces perihemorrhagic edema after intracerebral hemorrhage. Stroke (2010) 41:1684–9. 10.1161/STROKEAHA.110.587758 20616317

[60] Thomas Jefferson University. Targeted Temperature Management after Intracerebral Hemorrhage. Bethesda, MD: National Library of Medicine (US) (2000). Available from: https://clinicaltrials.gov/show/NCT01866384

[61] Koeppen AH Dickson AC Smith J. Heme oxygenase in experimental intracerebral hemorrhage: the benefit of tin-mesoporphyrin. J Neuropathol Exp Neurol (2004) 63:587–97. 10.1093/jnen/63.6.587 15217087

[62] Wang G Hu W Tang Q Wang L Sun XG Chen Y Effect comparison of both iron chelators on outcomes, iron deposit, and iron transporters after intracerebral hemorrhage in rats. Mol Neurobiol (2016) 53:3576–85. 10.1007/s12035-015-9302-3 26099311

[63] Ni W Zheng M Xi G Keep RF Hua Y. Role of lipocalin-2 in brain injury after intracerebral hemorrhage. J Cereb Blood Flow Metab (2015) 35:1454–61. 10.1038/jcbfm.2015.52 25853903 PMC4640334

[64] Wu J Hua Y Keep RF Nakamura T Hoff JT Xi G. Iron and iron-handling proteins in the brain after intracerebral hemorrhage. Stroke (2003) 34:2964–9. 10.1161/01.STR.0000103140.52838.45 14615611

[65] Mehdiratta M Kumar S Hackney D Schlaug G Selim M. Association between serum ferritin level and perihematoma edema volume in patients with spontaneous intracerebral hemorrhage. Stroke (2008) 39:1165–70. 10.1161/STROKEAHA.107.501213 18292378

[66] Aghaei I Bakhshayesh B Ramezani H Moosazadeh M Shabani M. The relationship between the serum levels of ferritin and the radiological brain injury indices in patients with spontaneous intracerebral hemorrhage. Iran J Basic Med Sci (2014) 17:729–34. 25729539 PMC4340978

[67] Pérez de la Ossa N Sobrino T Silva Y Blanco M Millán M Gomis M Iron-related brain damage in patients with intracerebral hemorrhage. Stroke (2010) 41:810–3. 10.1161/STROKEAHA.109.570168 20185788

[68] Yang G Hu R Zhang C Qian C Luo QQ Yung WH A combination of serum iron, ferritin and transferrin predicts outcome in patients with intracerebral hemorrhage. Sci Rep (2016) 6:21970. 10.1038/srep21970 26898550 PMC4761997

[69] Bakhshayesh B Hosseininezhad M Saadat SN Ansar MM Ramezani H Saadat SM. Iron overload is associated with perihematoma edema growth following intracerebral hemorrhage that may contribute to in-hospital mortality and long-term functional outcome. Curr Neurovasc Res (2014) 11:248–53. 10.2174/1567202611666140530124855 24875488

[70] Lou M Lieb K Selim M. The relationship between hematoma iron content and perihematoma edema: an MRI study. Cerebrovasc Dis (2009) 27:266–71. 10.1159/000199464 19202331 PMC2914447

[71] Hatakeyama T Okauchi M Hua Y Keep RF Xi G. Deferoxamine reduces neuronal death and hematoma lysis after intracerebral hemorrhage in aged rats. Transl Stroke Res (2013) 4:546–53. 10.1007/s12975-013-0270-5 24187595 PMC3810989

[72] Hatakeyama T Okauchi M Hua Y Keep RF Xi G. Deferoxamine reduces cavity size in the brain after intracerebral hemorrhage in aged rats. Acta Neurochir Suppl (2011) 111:185–90. 10.1007/978-3-7091-0693-8_31 21725753

[73] Gu Y Hua Y Keep RF Morgenstern LB Xi G. Deferoxamine reduces intracerebral hematoma-induced iron accumulation and neuronal death in piglets. Stroke (2009) 40:2241–3. 10.1161/STROKEAHA.108.539536 19372448 PMC2693321

[74] Hua Y Nakamura T Keep RF Wu J Schallert T Hoff JT Long-term effects of experimental intracerebral hemorrhage: the role of iron. J Neurosurg (2006) 104:305–12. 10.3171/jns.2006.104.2.305 16509506

[75] Selim M Yeatts S Goldstein JN Gomes J Greenberg S Morgenstern LB Safety and tolerability of deferoxamine mesylate in patients with acute intracerebral hemorrhage. Stroke (2011) 42:3067–74. 10.1161/STROKEAHA.111.617589 21868742 PMC3202043

[76] Yu Y Zhao W Zhu C Kong Z Xu Y Liu G The clinical effect of deferoxamine mesylate on edema after intracerebral hemorrhage. PLoS One (2015) 10:e0122371. 10.1371/journal.pone.0122371 25875777 PMC4395224

[77] National Institute of Neurological Disorders and Stroke, Beth Israel Deaconess Medical Center. Intracerebral Hemorrhage Deferoxamine Trial – iDEF Ttrial. Bethesda, MD: National Library of Medicine (US) (2000). Available from: https://clinicaltrials.gov/show/NCT02175225

[78] Capital Medical University. Deferoxamine and Xingnaojing Injection Treatment in Intracerebral Hemorrhage. Bethesda, MD: National Library of Medicine (US) (2000). Available from: https://clinicaltrials.gov/show/NCT02367248

[79] Chen-Roetling J Chen L Regan RF. Minocycline attenuates iron neurotoxicity in cortical cell cultures. Biochem Biophys Res Commun (2009) 386:322–6. 10.1016/j.bbrc.2009.06.026 19523448 PMC2782944

[80] Hess DC Fagan SC. Repurposing an old drug to improve the use and safety of tissue plasminogen activator for acute ischemic stroke: minocycline. Rev Neurol Dis (2010) 7(Suppl 1):S7–13. 20410869 PMC4730954

[81] Lee CZ Xue Z Zhu Y Yang GY Young WL. Matrix metalloproteinase-9 inhibition attenuates vascular endothelial growth factor-induced intracerebral hemorrhage. Stroke (2007) 38:2563–8. 10.1161/STROKEAHA.106.481515 17673717

[82] Wu J Yang S Hua Y Liu W Keep RF Xi G. Minocycline attenuates brain edema, brain atrophy and neurological deficits after intracerebral hemorrhage. Acta Neurochir Suppl (2010) 106:147–50. 10.1007/978-3-211-98811-4_26 19812938

[83] Zhao F Xi G Liu W Keep RF Hua Y. Minocycline attenuates iron-induced brain injury. Acta Neurochir Suppl (2016) 121:361–5. 10.1007/978-3-319-18497-5_62 26463975

[84] American Heart Association, Augusta University. A Pilot Study of Minocycline in Intracerebral Hemorrhage Patients (MACH). Bethesda, MD: National Library of Medicine (US) (2000). Available from: https://clinicaltrials.gov/show/NCT01805895

[85] Yang D Zhang J Han Y James E Chopp M Seyfried DM. Acute statin treatment improves recovery after experimental intracerebral hemorrhage. World J Neurosci (2013) 3:69–75. 10.4236/wjns.2013.32010 23837132 PMC3702179

[86] Yang D Knight RA Han Y Karki K Zhang J Ding C Vascular recovery promoted by atorvastatin and simvastatin after experimental intracerebral hemorrhage: magnetic resonance imaging and histological study. J Neurosurg (2011) 114:1135–42. 10.3171/2010.7.JNS10163 20722611 PMC3150838

[87] Jung KH Chu K Jeong SW Han SY Lee ST Kim JY HMG-CoA reductase inhibitor, atorvastatin, promotes sensorimotor recovery, suppressing acute inflammatory reaction after experimental intracerebral hemorrhage. Stroke (2004) 35:1744–9. 10.1161/01.STR.0000131270.45822.85 15166393

[88] Yang D Han Y Zhang J Chopp M Seyfried DM. Statins enhance expression of growth factors and activate the PI3K/Akt-mediated signaling pathway after experimental intracerebral hemorrhage. World J Neurosci (2012) 2:74–80. 10.4236/wjns.2012.22011 23482588 PMC3589759

[89] Cui JJ Wang D Gao F Li YR. Effects of atorvastatin on pathological changes in brain tissue and plasma MMP-9 in rats with intracerebral hemorrhage. Cell Biochem Biophys (2012) 62:87–90. 10.1007/s12013-011-9264-7 22006254

[90] Ewen T Qiuting L Chaogang T Tao T Jun W Liming T Neuroprotective effect of atorvastatin involves suppression of TNF-alpha and upregulation of IL-10 in a rat model of intracerebral hemorrhage. Cell Biochem Biophys (2013) 66:337–46. 10.1007/s12013-012-9453-z 23090788

[91] Seyfried D Han Y Lu D Chen J Bydon A Chopp M. Improvement in neurological outcome after administration of atorvastatin following experimental intracerebral hemorrhage in rats. J Neurosurg (2004) 101:104–7. 10.3171/jns.2004.101.1.0104 15255259

[92] Yang D Knight RA Han Y Karki K Zhang J Chopp M Statins protect the blood brain barrier acutely after experimental intracerebral hemorrhage. J Behav Brain Sci (2013) 3:100–6. 10.4236/jbbs.2013.31010 23459792 PMC3583226

[93] Karki K Knight RA Han Y Yang D Zhang J Ledbetter KA Simvastatin and atorvastatin improve neurological outcome after experimental intracerebral hemorrhage. Stroke (2009) 40:3384–9. 10.1161/STROKEAHA.108.544395 19644071 PMC2758425

[94] Guo X Bu X Jiang J Cheng P Yan Z. Enhanced neuroprotective effects of co-administration of G-CSF with simvastatin on intracerebral hemorrhage in rats. Turk Neurosurg (2012) 22:732–9. 10.5137/1019-5149.JTN.6177-12.1 23208905

[95] Chun HJ Kim DW Yi HJ Kim YS Kim EH Hwang SJ Effects of statin and deferoxamine administration on neurological outcomes in a rat model of intracerebral hemorrhage. Neurol Sci (2012) 33:289–96. 10.1007/s10072-011-0733-y 21863269

[96] Gioia LC Kate M McCourt R Gould B Coutts SB Dowlatshahi D Perihematoma cerebral blood flow is unaffected by statin use in acute intracerebral hemorrhage patients. J Cereb Blood Flow Metab (2015) 35:1175–80. 10.1038/jcbfm.2015.36 25757757 PMC4640272

[97] Ricard G Garant MP Carrier N Leblanc N Boulanger JM. Statins may increase intracerebral hemorrhage volume. Can J Neurol Sci (2010) 37:791–6. 10.1017/S0317167100051453 21059540

[98] Tapia-Perez H Sanchez-Aguilar M Torres-Corzo JG Rodriguez-Leyva I Gonzalez-Aguirre D Gordillo-Moscoso A Use of statins for the treatment of spontaneous intracerebral hemorrhage: results of a pilot study. Cen Eur Neurosurg (2009) 70:15–20. 10.1055/s-0028-1082064 19197830

[99] Tapia Perez JH Yildiz OC Schneider T Nimsky C. Meta-analysis of statin use for the acute therapy of spontaneous intracerebral hemorrhage. J Stroke Cerebrovasc Dis (2015) 24:2521–6. 10.1016/j.jstrokecerebrovasdis.2015.06.036 26387046

[100] Tapia-Perez JH Zilke R Schneider T. Match-study of statin therapy in spontaneous intracerebral hemorrhage: is the discontinuation reasonable? J Neurosurg Sci (2016) 60:301–12. 25501007

[101] Tapia-Perez JH Rupa R Zilke R Gehring S Voellger B Schneider T. Continued statin therapy could improve the outcome after spontaneous intracerebral hemorrhage. Neurosurg Rev (2013) 36:279–87; discussion 87. 10.1007/s10143-012-0431-0 23097148

[102] Dowlatshahi D Demchuk AM Fang J Kapral MK Sharma M Smith EE Association of statins and statin discontinuation with poor outcome and survival after intracerebral hemorrhage. Stroke (2012) 43:1518–23. 10.1161/STROKEAHA.111.645978 22442172

[103] Lee ST Chu K Jung KH Kim J Kim EH Kim SJ Memantine reduces hematoma expansion in experimental intracerebral hemorrhage, resulting in functional improvement. J Cereb Blood Flow Metab (2006) 26:536–44. 10.1038/sj.jcbfm.9600213 16107786

[104] Sinn DI Lee ST Chu K Jung KH Song EC Kim JM Combined neuroprotective effects of celecoxib and memantine in experimental intracerebral hemorrhage. Neurosci Lett (2007) 411:238–42. 10.1016/j.neulet.2006.10.050 17123715

[105] Bakhshayesh-Eghbali BHM Seyed-Saadat SM Seyed-Saadat SN Kazemnezhad-Leili E Rouhi-Rad M Comparing the effect of memantine and placebo on clinical outcome of intracranial hemorrhage: a randomized double blind clinical trial. Caspian J Neurol Sci (2015) 1:11–8.

[106] Chu K Jeong SW Jung KH Han SY Lee ST Kim M Celecoxib induces functional recovery after intracerebral hemorrhage with reduction of brain edema and perihematomal cell death. J Cereb Blood Flow Metab (2004) 24:926–33. 10.1097/01.WCB.0000130866.25040.7D 15362723

[107] Park HK Lee SH Chu K Roh JK. Effects of celecoxib on volumes of hematoma and edema in patients with primary intracerebral hemorrhage. J Neurol Sci (2009) 279:43–6. 10.1016/j.jns.2008.12.020 19168192

[108] Lee SH Park HK Ryu WS Lee JS Bae HJ Han MK Effects of celecoxib on hematoma and edema volumes in primary intracerebral hemorrhage: a multicenter randomized controlled trial. Eur J Neurol (2013) 20:1161–9. 10.1111/ene.12140 23551657

[109] Gong C Hoff JT Keep RF. Acute inflammatory reaction following experimental intracerebral hemorrhage in rat. Brain Res (2000) 871:57–65. 10.1016/S0006-8993(00)02427-6 10882783

[110] Yang GY Betz AL Chenevert TL Brunberg JA Hoff JT. Experimental intracerebral hemorrhage: relationship between brain edema, blood flow, and blood-brain barrier permeability in rats. J Neurosurg (1994) 81:93–102. 10.3171/jns.1994.81.1.0093 8207532

[111] Schlunk F Pfeilschifter W Yigitkanli K Lo EH Foerch C. Treatment with FTY720 has no beneficial effects on short-term outcome in an experimental model of intracerebral hemorrhage. Exp Transl Stroke Med (2016) 8:1. 10.1186/s13231-016-0016-z 26893821 PMC4758011

[112] Lu L Barfejani AH Qin T Dong Q Ayata C Waeber C. Fingolimod exerts neuroprotective effects in a mouse model of intracerebral hemorrhage. Brain Res (2014) 1555:89–96. 10.1016/j.brainres.2014.01.048 24502984 PMC3994537

[113] Rolland WB Lekic T Krafft PR Hasegawa Y Altay O Hartman R Fingolimod reduces cerebral lymphocyte infiltration in experimental models of rodent intracerebral hemorrhage. Exp Neurol (2013) 241:45–55. 10.1016/j.expneurol.2012.12.009 23261767 PMC3570752

[114] Rolland WB II Manaenko A Lekic T Hasegawa Y Ostrowski R Tang J FTY720 is neuroprotective and improves functional outcomes after intracerebral hemorrhage in mice. Acta Neurochir Suppl (2011) 111:213–7. 10.1007/978-3-7091-0693-8_36 21725758 PMC3569072

[115] Li YJ Chang GQ Liu Y Gong Y Yang C Wood K Fingolimod alters inflammatory mediators and vascular permeability in intracerebral hemorrhage. Neurosci Bull (2015) 31:755–62. 10.1007/s12264-015-1532-2 25958190 PMC5563722

[116] Fu Y Hao J Zhang N Ren L Sun N Li YJ Fingolimod for the treatment of intracerebral hemorrhage: a 2-arm proof-of-concept study. JAMA Neurol (2014) 71:1092–101. 10.1001/jamaneurol.2014.1065 25003359

[117] Sun N Shen Y Han W Shi K Wood K Fu Y Selective sphingosine-1-phosphate receptor 1 modulation attenuates experimental intracerebral hemorrhage. Stroke (2016) 47:1899–906. 10.1161/STROKEAHA.115.012236 27174529

[118] Song EC Chu K Jeong SW Jung KH Kim SH Kim M Hyperglycemia exacerbates brain edema and perihematomal cell death after intracerebral hemorrhage. Stroke (2003) 34:2215–20. 10.1161/01.STR.0000088060.83709.2C 12907821

[119] Lin X Tang Y Sun B Hou Z Meng H Li Z Cerebral glucose metabolism: influence on perihematomal edema formation after intracerebral hemorrhage in cat models. Acta Radiol (2010) 51:549–54. 10.3109/02841851003660065 20307247

[120] Qureshi AI Palesch YY Martin R Novitzke J Cruz-Flores S Ehtisham A Association of serum glucose concentrations during acute hospitalization with hematoma expansion, perihematomal edema, and three month outcome among patients with intracerebral hemorrhage. Neurocrit Care (2011) 15:428–35. 10.1007/s12028-011-9541-8 21573860

[121] Feng W Tauhid S Goel S Sidorov EV Selim M. Hyperglycemia and outcome in intracerebral hemorrhage: from bedside to bench-more study is needed. Transl Stroke Res (2012) 3:113–8. 10.1007/s12975-012-0163-z 24323866

[122] Mayer SA Brun NC Broderick J Davis S Diringer MN Skolnick BE Safety and feasibility of recombinant factor VIIa for acute intracerebral hemorrhage. Stroke (2005) 36:74–9. 10.1161/01.STR.0000149628.80251.b8 15569871

[123] Demchuk AM Dowlatshahi D Rodriguez-Luna D Molina CA Blas YS Dzialowski I Prediction of haematoma growth and outcome in patients with intracerebral haemorrhage using the CT-angiography spot sign (PREDICT): a prospective observational study. Lancet Neurol (2012) 11:307–14. 10.1016/S1474-4422(12)70038-8 22405630

[124] Barras CD Tress BM Christensen S MacGregor L Collins M Desmond PM Density and shape as CT predictors of intracerebral hemorrhage growth. Stroke (2009) 40:1325–31. 10.1161/STROKEAHA.108.536888 19286590

[125] Kawai N Nakamura T Nagao S. Early hemostatic therapy using recombinant factor VIIa in a collagenase-induced intracerebral hemorrhage model in rats. Acta Neurochir Suppl (2006) 96:212–7. 10.1007/3-211-30714-1_46 16671457

[126] Arkin S Cooper HA Hutter JJ Miller S Schmidt ML Seibel NL Activated recombinant human coagulation factor VII therapy for intracranial hemorrhage in patients with hemophilia A or B with inhibitors. Results of the novoseven emergency-use program. Haemostasis (1998) 28:93–8. 10087434 10.1159/000022418

[127] Mayer SA Brun NC Begtrup K Broderick J Davis S Diringer MN Recombinant activated factor VII for acute intracerebral hemorrhage. N Engl J Med (2005) 352:777–85. 10.1056/NEJMoa042991 15728810

[128] Mayer SA Brun NC Begtrup K Broderick J Davis S Diringer MN Efficacy and safety of recombinant activated factor VII for acute intracerebral hemorrhage. N Engl J Med (2008) 358:2127–37. 10.1056/NEJMoa0707534 18480205

[129] Sutherland CS Hill MD Kaufmann AM Silvaggio JA Demchuk AM Sutherland GR. Recombinant factor VIIa plus surgery for intracerebral hemorrhage. Can J Neurol Sci (2008) 35:567–72. 10.1017/S0317167100009343 19235439

[130] Imberti R Pietrobono L Klersy C Gamba G Iotti G Cornara G. Intraoperative intravenous administration of rFVIIa and hematoma volume after early surgery for spontaneous intracerebral hemorrhage: a randomized prospective phase II study. Minerva Anestesiol (2012) 78:168–75. 21750485

[131] Frontera JA Lewin JJ III Rabinstein AA Aisiku IP Alexandrov AW Cook AM Guideline for reversal of antithrombotics in intracranial hemorrhage. Neurocrit Care (2016) 24:6–46. 10.1007/s12028-015-0222-x 26714677

[132] Illanes S Zhou W Heiland S Markus Z Veltkamp R. Kinetics of hematoma expansion in murine warfarin-associated intracerebral hemorrhage. Brain Res (2010) 1320:135–42. 10.1016/j.brainres.2010.01.015 20085756

[133] Schlunk F Schulz E Lauer A Yigitkanli K Pfeilschifter W Steinmetz H Warfarin pretreatment reduces cell death and MMP-9 activity in experimental intracerebral hemorrhage. Transl Stroke Res (2015) 6:133–9. 10.1007/s12975-014-0377-3 25424451

[134] Brouwers HB Chang Y Falcone GJ Cai X Ayres AM Battey TW Predicting hematoma expansion after primary intracerebral hemorrhage. JAMA Neurol (2014) 71:158–64. 10.1001/jamaneurol.2013.5433 24366060 PMC4131760

[135] Okada T Nakase T Sasaki M Ishikawa T. Do the antithrombotic therapy at the time of intracerebral hemorrhage influence clinical outcome? analysis between the difference of antiplatelet and anticoagulant agents and clinical course. J Stroke Cerebrovasc Dis (2014) 23:1781–8. 10.1016/j.jstrokecerebrovasdis.2014.04.036 24957306

[136] Takahashi H Jimbo Y Takano H Abe H Sato M Fujii Y Intracerebral hematoma occurring during warfarin versus non-vitamin K antagonist oral anticoagulant therapy. Am J Cardiol (2016) 118:222–5. 10.1016/j.amjcard.2016.04.034 27289294

[137] Yaghi S Dibu J Achi E Patel A Samant R Hinduja A. Hematoma expansion in spontaneous intracerebral hemorrhage: predictors and outcome. Int J Neurosci (2014) 124:890–3. 10.3109/00207454.2014.887716 24472073

[138] Chai-Adisaksopha C Hillis C Siegal DM Movilla R Heddle N Iorio A Prothrombin complex concentrates versus fresh frozen plasma for warfarin reversal. A systematic review and meta-analysis. Thromb Haemost (2016) 116(5):879–90. 10.1160/TH16-04-0266 27488143

[139] University of Utah. FFP versus PCC in Intracranial Hemorrhage. Bethesda, MD: National Library of Medicine (US) (2000). Available from: https://clinicaltrials.gov/show/NCT02429453

[140] Mittal MK Rabinstein AA. Anticoagulation-related intracranial hemorrhages. Curr Atheroscler Rep (2012) 14:351–9. 10.1007/s11883-012-0258-8 22638877

[141] Lauer A Cianchetti FA Van Cott EM Schlunk F Schulz E Pfeilschifter W Anticoagulation with the oral direct thrombin inhibitor dabigatran does not enlarge hematoma volume in experimental intracerebral hemorrhage. Circulation (2011) 124:1654–62. 10.1161/CIRCULATIONAHA.111.035972 21911784 PMC3724228

[142] Akiyama H Uchino K Hasegawa Y. Characteristics of symptomatic intracranial hemorrhage in patients receiving non-vitamin K antagonist oral anticoagulant therapy. PLoS One (2015) 10:e0132900. 10.1371/journal.pone.0132900 26171862 PMC4501739

[143] Hagii J Tomita H Metoki N Saito S Shiroto H Hitomi H Characteristics of intracerebral hemorrhage during rivaroxaban treatment: comparison with those during warfarin. Stroke (2014) 45:2805–7. 10.1161/STROKEAHA.114.006661 25082810

[144] Simonsen CZ Steiner T Tietze A Damgaard D. Dabigatran-related intracerebral hemorrhage resulting in hematoma expansion. J Stroke Cerebrovasc Dis (2014) 23:e133–4. 10.1016/j.jstrokecerebrovasdis.2013.08.011 24103671

[145] Pollack CV Jr Reilly PA Eikelboom J Glund S Verhamme P Bernstein RA Idarucizumab for dabigatran reversal. N Engl J Med (2015) 373:511–20. 10.1056/NEJMoa1502000 26095746

[146] Na SY Mracsko E van Ryn J Veltkamp R. Idarucizumab improves outcome in murine brain hemorrhage related to dabigatran. Ann Neurol (2015) 78:137–41. 10.1002/ana.24421 25899749

[147] Connolly SJ Milling TJ Jr Eikelboom JW Gibson CM Curnutte JT Gold A Andexanet alfa for acute major bleeding associated with factor Xa inhibitors. N Engl J Med (2016) 375:1131–41. 10.1056/NEJMoa1607887 27573206 PMC5568772

[148] UMC Utrecht. Apixaban versus Antiplatelet Drugs or No Antithrombotic Drugs after Anticoagulation-Associated Intracerebral Haemorrhage in Patients with Atrial Fibrillation (APACHE-AF). Bethesda, MD: National Library of Medicine (US) (2000). Available from: https://clinicaltrials.gov/show/NCT02565693 10.1186/s13063-015-0898-4 PMC4560912 26340977

[149] Lauer A Schlunk F Van Cott EM Steinmetz H Lo EH Foerch C Antiplatelet pretreatment does not increase hematoma volume in experimental intracerebral hemorrhage. J Cereb Blood Flow Metab (2011) 31:1736–42. 10.1038/jcbfm.2011.22 21386857 PMC3170939

[150] Campbell PG Yadla S Sen AN Jallo J Jabbour P. Emergency reversal of clopidogrel in the setting of spontaneous intracerebral hemorrhage. World Neurosurg (2011) 76:100–4; discussion 59–60. 10.1016/j.wneu.2011.02.010 21839960

[151] Naidech AM Maas MB Levasseur-Franklin KE Liotta EM Guth JC Berman M Desmopressin improves platelet activity in acute intracerebral hemorrhage. Stroke (2014) 45:2451–3. 10.1161/STROKEAHA.114.006061 25005444 PMC6211162

[152] Baharoglu MI Cordonnier C Al-Shahi Salman R de Gans K Koopman MM Brand A Platelet transfusion versus standard care after acute stroke due to spontaneous cerebral haemorrhage associated with antiplatelet therapy (PATCH): a randomised, open-label, phase 3 trial. Lancet (2016) 387:2605–13. 10.1016/S0140-6736(16)30392-0 27178479

[153] Qureshi AI Acute hypertensive response in patients with stroke: pathophysiology and management. Circulation (2008) 118:176–87. 10.1161/CIRCULATIONAHA.107.723874 18606927

[154] Sang YH Su HX Wu WT So KF Cheung RT. Elevated blood pressure aggravates intracerebral hemorrhage-induced brain injury. J Neurotrauma (2011) 28:2523–34. 10.1089/neu.2010.1680 21988112 PMC3235342

[155] Benveniste H Kim KR Hedlund LW Kim JW Friedman AH. Cerebral hemorrhage and edema following brain biopsy in rats: significance of mean arterial blood pressure. J Neurosurg (2000) 92:100–7. 10.3171/jns.2000.92.1.0100 10616088

[156] Sansing LH Messe SR Cucchiara BL Lyden PD Kasner SE. Anti-adrenergic medications and edema development after intracerebral hemorrhage. Neurocrit Care (2011) 14:395–400. 10.1007/s12028-010-9498-z 21264527

[157] Kalita J Misra UK Kumar B Is beta-blocker (atenolol) a preferred antihypertensive in acute intracerebral hemorrhage? Neurol Sci (2013) 34:1099–104. 10.1007/s10072-012-1210-y 23053838

[158] Sundbøll J Schmidt M Horváth-Puhó E Christiansen CF Pedersen L Bøtker HE Impact of preadmission treatment with calcium channel blockers or beta blockers on short-term mortality after stroke: a nationwide cohort study. BMC Neurol (2015) 15:24. 10.1186/s12883-015-0279-3 25884780 PMC4365558

[159] Shoup JP Winkler J Czap A Staff I Fortunato G McCullough LD beta-Blockers associated with no class-specific survival benefit in acute intracerebral hemorrhage. J Neurol Sci (2014) 336:127–31. 10.1016/j.jns.2013.10.022 24183854 PMC4956481

[160] Qureshi AI Palesch YY Barsan WG Hanley DF Hsu CY Martin RL Intensive blood-pressure lowering in patients with acute cerebral hemorrhage. N Engl J Med (2016) 375:1033–43. 10.1056/NEJMoa1603460 27276234 PMC5345109

[161] Anderson CS Heeley E Huang Y Wang J Stapf C Delcourt C Rapid blood-pressure lowering in patients with acute intracerebral hemorrhage. N Engl J Med (2013) 368:2355–65. 10.1056/NEJMoa1214609 23713578

[162] Ma J Li H Liu Y You C Huang S Ma L. Effects of intensive blood pressure lowering on intracerebral hemorrhage outcomes: a meta-analysis of randomized controlled trials. Turk Neurosurg (2015) 25:544–51. 10.5137/1019-5149.JTN.9270-13.0 26242330

[163] University of California, Los Angeles. Field Administration of Stroke Therapy-Blood Pressure Lowering (FAST-BP). Bethesda, MD: National Library of Medicine (US) (2000). Available from: https://clinicaltrials.gov/show/NCT01811693

[164] Fujimoto S Katsuki H Ohnishi M Takagi M Kume T Akaike A. Plasminogen potentiates thrombin cytotoxicity and contributes to pathology of intracerebral hemorrhage in rats. J Cereb Blood Flow Metab (2008) 28:506–15. 10.1038/sj.jcbfm.9600547 17940541

[165] Arumugam A A Rahman NA Theophilus SC Shariffudin A Abdullah JM. Tranexamic acid as antifibrinolytic agent in non traumatic intracerebral hemorrhages. Malays J Med Sci (2015) 22:62–71. 27006639 PMC4795525

[166] Sprigg N Renton CJ Dineen RA Kwong Y Bath PM. Tranexamic acid for spontaneous intracerebral hemorrhage: a randomized controlled pilot trial (ISRCTN50867461). J Stroke Cerebrovasc Dis (2014) 23:1312–8. 10.1016/j.jstrokecerebrovasdis.2013.11.007 24680087

[167] Neuroscience Trials Australia, The Florey Institute of Neuroscience and Mental Health. STOP-AUST: The Spot Sign and Tranexamic Acid on Preventing ICH Growth – AUStralasia Trial (STOP-AUST). Bethesda, MD: National Library of Medicine (US) (2000). Available from: https://clinicaltrials.gov/show/NCT01702636

[168] Ministry of Science and Technology of the People’s Republic of China, Beijing Municipal Science & Technology Commission. Tranexamic Acid for Acute ICH Growth Predicted by Spot Sign (TRAIGE). Bethesda, MD: National Library of Medicine (US) (2000). Available from: https://clinicaltrials.gov/show/NCT02625948

[169] Liu J Gao BB Clermont AC Blair P Chilcote TJ Sinha S Hyperglycemia-induced cerebral hematoma expansion is mediated by plasma kallikrein. Nat Med (2011) 17:206–10. 10.1038/nm.2295 21258336 PMC3038677

[170] Matsushita H Hijioka M Hisatsune A Isohama Y Iwamoto S Terasawa H MRI-based analysis of intracerebral hemorrhage in mice reveals relationship between hematoma expansion and the severity of symptoms. PLoS One (2013) 8:e67691. 10.1371/journal.pone.0067691 23844065 PMC3699642

